# Biallelic pathogenic variants in *POLR3D* alter tRNA transcription and cause a hypomyelinating leukodystrophy: A case report

**DOI:** 10.3389/fneur.2023.1254140

**Published:** 2023-10-13

**Authors:** Julia Macintosh, Stefanie Perrier, Maxime Pinard, Luan T. Tran, Kether Guerrero, Chitra Prasad, Asuri N. Prasad, Tomi Pastinen, Isabelle Thiffault, Benoit Coulombe, Geneviève Bernard

**Affiliations:** ^1^Department of Neurology and Neurosurgery, McGill University, Montreal, QC, Canada; ^2^Child Health and Human Development Program, Research Institute of the McGill University Health Centre, Montreal, QC, Canada; ^3^Institut de Recherches Cliniques de Montréal, Montreal, QC, Canada; ^4^Department of Pediatrics, London Health Sciences Center and Western University, London, ON, Canada; ^5^Medical Genetics Program of Southwestern Ontario, London Health Sciences Center, London, ON, Canada; ^6^Children’s Health Research Institute, London, ON, Canada; ^7^Genomic Medicine Center, Children's Mercy Hospital, Kansas City, MO, United States; ^8^University of Missouri Kansas City School of Medicine, Kansas City, MO, United States; ^9^Department of Pediatrics, Children's Mercy Hospital, Kansas City, MO, United States; ^10^Department of Pathology and Laboratory Medicine, Children's Mercy Hospital, Kansas City, MO, United States; ^11^Département de Biochimie et Médecine Moléculaire, Faculté de Médecine, Université de Montréal, Montreal, QC, Canada; ^12^Department of Pediatrics, McGill University, Montreal, QC, Canada; ^13^Department of Human Genetics, McGill University, Montreal, QC, Canada; ^14^Department of Specialized Medicine, Division of Medical Genetics, McGill University Health Centre, Montreal, QC, Canada

**Keywords:** POLR3-related leukodystrophy, 4H leukodystrophy, hypomyelination, RNA polymerase III, *POLR3D*, RPC4

## Abstract

RNA polymerase III-related leukodystrophy (POLR3-related leukodystrophy) is a rare, genetically determined hypomyelinating disease arising from biallelic pathogenic variants in genes encoding subunits of RNA polymerase III (Pol III). Here, we describe the first reported case of POLR3-related leukodystrophy caused by biallelic pathogenic variants in *POLR3D*, encoding the RPC4 subunit of Pol III. The individual, a female, demonstrated delays in walking and expressive and receptive language as a child and later cognitively plateaued. Additional neurological features included cerebellar signs (e.g., dysarthria, ataxia, and intention tremor) and dysphagia, while non-neurological features included hypodontia, hypogonadotropic hypogonadism, and dysmorphic facial features. Her MRI was notable for diffuse hypomyelination with myelin preservation of early myelinating structures, characteristic of POLR3-related leukodystrophy. Exome sequencing revealed the biallelic variants in *POLR3D*, a missense variant (c.541C > T, p.P181S) and an intronic splice site variant (c.656-6G > A, p.?). Functional studies of the patient’s fibroblasts demonstrated significantly decreased RNA-level expression of *POLR3D*, along with reduced expression of other Pol III subunit genes. Notably, Pol III transcription was also shown to be aberrant, with a significant decrease in *7SK* RNA and several distinct tRNA genes analyzed. Affinity purification coupled to mass spectrometry of the *POLR3D* p.P181S variant showed normal assembly of Pol III subunits yet altered interaction of Pol III with the PAQosome chaperone complex, indicating the missense variant is likely to alter complex maturation. This work identifies biallelic pathogenic variants in *POLR3D* as a novel genetic cause of POLR3-related leukodystrophy, expanding the molecular spectrum associated with this disease, and proposes altered tRNA homeostasis as a factor in the underlying biology of this hypomyelinating disorder.

## Introduction

Leukodystrophies are a heterogeneous group of rare, inherited white matter disorders (1). In particular, as one of the most common hypomyelinating leukodystrophies, POLR3-related leukodystrophy presents in previously healthy children with a variety of neurological and non-neurological clinical features, together with a characteristic hypomyelination pattern on brain MRI (2, 3). The MRI pattern includes hypomyelination, appearing as hyperintensity of white matter relative to gray matter on T2-weighted images and variable signal (hyperintense, hypointense, or isointense) of white matter relative to gray matter on T1-weighted images, along with relative preservation of myelination in early myelinating structures (i.e., dentate nucleus, optic radiations, globus pallidus, anterolateral nucleus of the thalamus, and in some cases, corticospinal tracts at the level of the posterior limb of the internal capsule), with or without thinning of the corpus callosum and cerebellar atrophy (4). Neurological features are predominantly motor and can include cerebellar (e.g., abnormal extraocular movements, dysarthria, ataxia, and dysmetria), pyramidal (e.g., spasticity), and extrapyramidal (e.g., dystonia) signs, along with variable cognitive involvement (3). POLR3-related leukodystrophy is further clinically distinguished from other leukodystrophies by characteristic non-neurological features including dental abnormalities (e.g., hypodontia) and endocrine abnormalities (e.g., hypogonadotropic hypogonadism and short stature), from which the alternate disease name, 4H leukodystrophy (i.e., hypomyelination, hypodontia, and hypogonadotropic hypogonadism) was derived (5). Ocular abnormalities, typically myopia, are also seen, as is craniofacial involvement, the latter most commonly seen in individuals harboring biallelic pathogenic variants in the *POLR1C* gene (5–7).

RNA polymerase III (Pol III) is a 17-subunit enzymatic complex tasked, in eukaryotes, with the transcription of critical and ubiquitous non-coding RNAs, including 5S ribosomal RNA, all tRNAs, and a variety of other non-coding RNAs involved in transcription, RNA processing and localization, and/or translation (8). While autosomal recessive variants in *POLR3A* (OMIM *614258; OMIM #607694) and *POLR3B* (OMIM *614366; OMIM #614381), which encode the catalytic subunits of the Pol III enzymatic complex, were originally identified as the etiology of this disease, biallelic pathogenic variants in *POLR1C* (OMIM *610060; OMIM #616494) and *POLR3K* (OMIM *606007; OMIM #619310) have since been recognized as causative (9–12). Nonetheless, a subset of individuals with clinical and neuroradiological characteristics aligning with this disease still remain without a genetic diagnosis.

In this study, we report the first case of POLR3-related leukodystrophy arising from biallelic pathogenic variants in the *POLR3D* gene, in an individual with a clinical and neuroradiological phenotype consistent with POLR3-related leukodystrophy. In addition, we demonstrate that these novel variants, including a missense variant [NM_001722.3 (*POLR3D*): c.541C > T, p.P181S] and an intronic splice site variant [NM_001722.3 (*POLR3D*): c.656-6G > A, p.?], cause altered RNA-level expression of Pol III subunits and Pol III transcripts. We also demonstrate that the missense variant impacts the interaction between Pol III and its chaperone complex.

## Methods

### Informed consent, clinical and neuroimaging review

Written informed consent from the legal representative of the patient was obtained. This study was approved by the Montreal Children’s Hospital and the McGill University Health Center Research Ethic Boards (11-105-PED, 2019-4972) and the Children’s Mercy Institutional Review Board (study #11120514). A retrospective review of available medical records and brain MRI was performed.

### Genetic analysis

Using genomic DNA extracted from peripheral blood leukocytes, exome sequencing was initially performed by PerkinElmer, as previously described (12), and later repeated using the Illumina TruSeq library preparation and IDT XGen Exome Enrichment protocol (13). Sequences were aligned to reference gene sequences based on the human genome build (GRCh38/hg38) and analyzed using the custom-developed software RUNES and Viking (14). Variant interpretation was performed using the American College of Medical Genetics and Genomics (ACMG) standards and guidelines for the interpretation of sequence variants (14).

### RT-qPCR

To analyze transcription levels of Pol III subunit genes and Pol III-transcribed genes, RNA was collected from fibroblasts and cDNA was synthesized for RT-qPCR analysis. Primer designs are provided in the supplemental materials (Supplementary Table S1).

### Plasmid construction and stable clones production

To assess how the missense variant in *POLR3D* impacts the assembly of the Pol III complex, the *POLR3D* WT sequence (NCBI sequence: BC004484) was amplified by PCR using the following primers (*POLR3D* HindIII F: 5′-CCC AAG CTT ATG TCG GAA GGA AAC GCC GCC G-3′ and *POLR3D* XhoI R: 5′-CCG CTC GAG TTA CCG GTG TTT GTG ATC CAA GAG G-3′) and cloned in the pCMV-3tag-1a parental plasmids (between HindIII and XhoI cloning site). The p.P181S mutation was introduced by site-directed mutagenesis by using the overlapping primers F: 5′-GAA ATA TGC CTG TGC AGC TGT CGC TGG CTC ACT CAG GAT G-3′ and R: 5′-CAT CCT GAG TGA GCC AGC GAC AGC TGC ACA GGC ATA TTT C-3′ (15). Both sequences were confirmed by Sanger sequencing. Stable clones of POLR3D WT and p.P181S were produced in HeLa cells by transfecting each digested plasmids with Jet Prime, following manufacturer’s instruction. 3x-FLAG-POLR3 WT and p.P181S expression level of the different clones was assessed via western blotting by using an anti-FLAG antibody to selected clones with equivalent expression.

### Affinity purification—mass spectrometry

Affinity purification followed by liquid chromatography–tandem mass spectrometry (LC–MS/MS) acquisition and raw data analysis were performed, as previously described (16).

Detailed methods pertaining to genetic analysis, fibroblast culture, RT-qPCR work, and proteomic studies are described in the Supplementary material.

## Results

### Clinical characteristics and early investigations

The patient, a female, was born to non-consanguineous parents. The pregnancy was complicated by concerns over fetal size throughout the gestational period and a large blood clot was passed in the first few weeks of pregnancy. Delivery was unremarkable. In the neonatal period, the patient was noted to have feeding difficulties and required IV fluids initially, which later improved. Her development was noted to be delayed and she did not walk until 18 months. In her early years of school, teachers noticed delays in both expressive and receptive language, and she was formally diagnosed with learning disabilities. Vision and hearing were normal. She experienced a cognitive plateau and at age 26 years, was functioning at a grade 6 reading level and grade 3 comprehension level. Neuropsychological assessment revealed that most areas of cognition, including working memory and attention, were impaired. She was also noted to have cerebellar involvement, including an ataxic gait, intention tremor, and dysarthria. Silent laryngeal aspirations of thin fluids were remarked on a modified barium swallow study, and she was diagnosed with dysphagia at age 28 years. In her late 20s, she started to have trouble with ambulation and became wheelchair dependent by her mid-30s, at which time she was also considered to be anarthric. She died at the age of 37.

Of interest, she also had various non-neurological features characteristic of POLR3-related leukodystrophy. These included hypodontia, as she was missing two maxillary lateral incisors and two mandibular second premolars. Regarding endocrine abnormalities, she had hypogonadotropic hypogonadism. On MRI, her pituitary gland was noted to be small. A bone mineral density exam revealed osteopenia. She did not have short stature nor ocular involvement, including no myopia. Interestingly, she was noted to have non-specific dysmorphic facial features, with deep-set eyes and frontal prominence.

Various investigations were pursued to identify the etiology of her neurodegenerative condition. Karyotyping and multi-telomere fluorescence *in situ* hybridization (FISH) revealed no chromosomal or telomere abnormalities, respectively. A negative transferrin isoelectric focusing ruled out disorders of glycosylation, and mitochondrial DNA analysis was unremarkable. Metabolic diseases, including mucopolysaccharidosis and mucolipidosis type IV, were considered but ruled out upon further testing. An expansion was found in the *SCA8* gene (95/25), inherited from the father (124/23), and associated with spinocerebellar ataxia type 8. However, given that expansions of *SCA8* are not all associated with cerebellar ataxia, and considering her age at disease onset and the presence of hypomyelination and non-neurological features, this finding was considered unlikely to explain her disease (17, 18). At age 24 years, a clinical diagnosis of 4H leukodystrophy was suggested, prior to the identification of the genetic etiology of this disorder (19).

### Imaging studies

**Figure 1:**
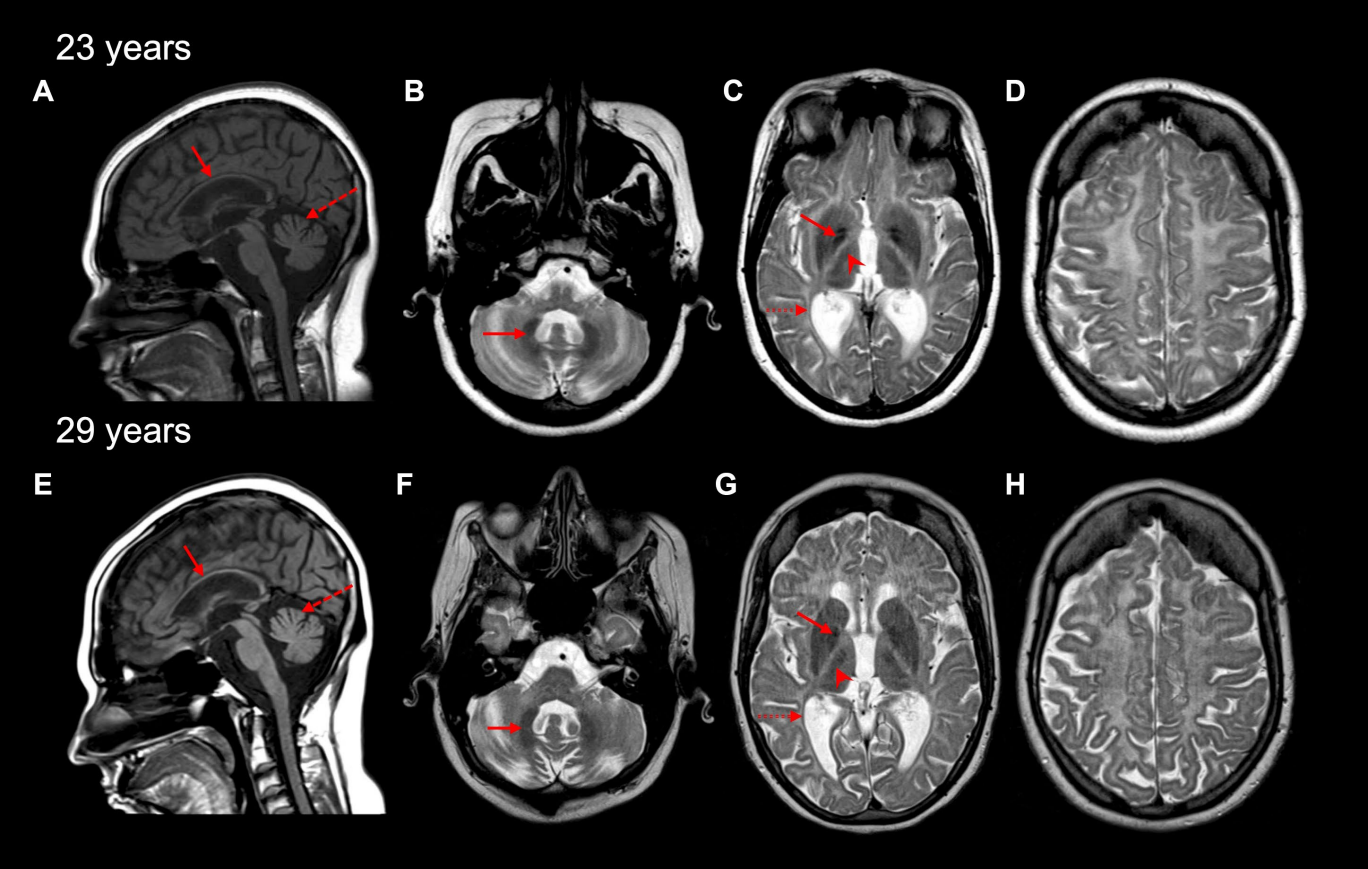
Brain MRI characteristics of the affected individual at 23 and 29 years old. A thin corpus callosum (red arrow) and cerebellar vermis atrophy (red dashed arrow) are seen on sagittal T1-weighted MRI images **(A,E)**. Cerebellar hemisphere atrophy was also seen at 23 and 29 years (not shown). Diffuse hypomyelination is evident **(C,D,G,H)**, with relative preservation of myelin seen in the dentate nucleus (red arrow in **B,F**), globus pallidus (red arrow in **C,G**), the anterolateral nucleus of the thalamus (red arrowhead in **C,G**), and optic radiations (dashed red arrow in **C,G**) on axial T2-weighted MRI images.

Initial magnetic resonance imaging (MRI) performed at age 17 years revealed diffuse hypomyelination. Repeat MRIs at 23 and 29 years likewise demonstrated these white matter abnormalities, along with a thin corpus callosum and cerebellar atrophy (Figures 1A,E). A pattern of myelin preservation was seen in certain structures, including the optic radiations, dentate nucleus, anterolateral nucleus of the thalamus, and the globus pallidus, which are known to be early-myelinating structures characteristically spared in POLR3-related leukodystrophy (Figure 1) (5, 20). Cerebellar atrophy and a thin corpus callosum were likewise seen on a computed tomography (CT) scan performed at age 24 years, which also revealed mild–moderate ventricular enlargement of both the infra- and supratentorial ventricles and cerebral atrophy.

### Exome sequencing identifies biallelic variants in *POLR3D*

**Figure 2:**
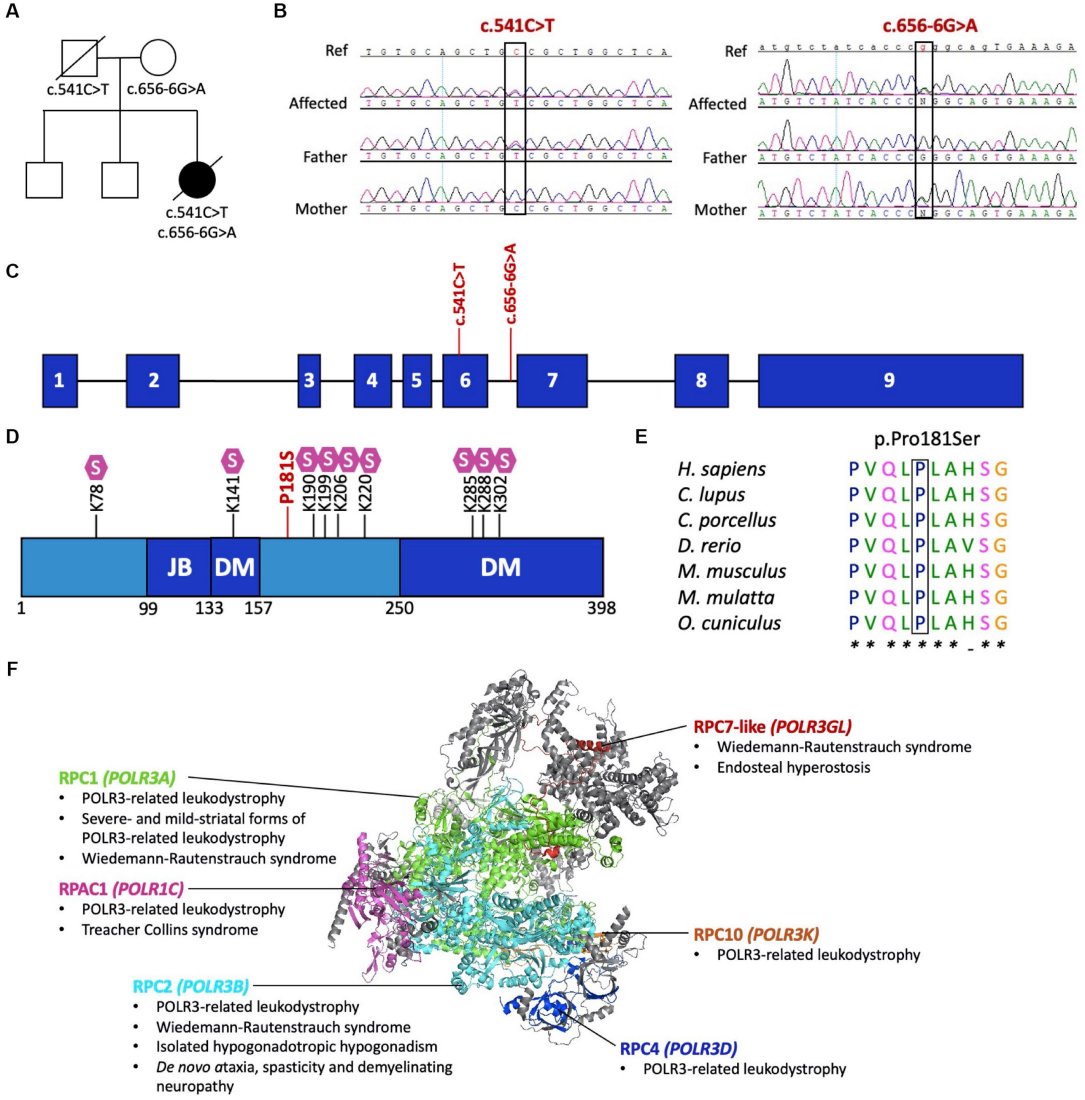
Pathogenic variants in *POLR3D*. **(A)** Familial segregation of the c.541C > T (p.P181S) and c.656-6G>A (p.?). **(B)** *POLR3D* sequence chromatograms of the affected individual and her parents show segregation of the variants. **(C)** Schematic of *POLR3D* genomic DNA with pathogenic variants mapped. **(D)** Schematic of the domain organization of the RPC4 protein; JB, Jaw binding; DM, Dimerization domain. Lysine residues subjected to sumoylation (S) are marked on the sequence. The missense mutation (red) found in the patient described here is proximal to a sumoylation hotspot. **(E)** The missense variant in *POLR3D* is at a conserved amino acid residue. **(F)** Modeled human structure of RNA polymerase III. Colors identify Pol III subunits encoded by genes that harbor pathogenic variants, including POLR3A (green), POLR3B (cyan), POLR1C (magenta), POLR3D (blue), POLR3K (orange), and POLR3GL (red). Phenotypes associated with the genes are listed under the protein name. In all cases inheritance is autosomal recessive, except for *de novo* ataxia, spasticity, and demyelinating neuropathy. Modeled on PyMOL, PDB: 7A6H.

Initial research exome sequencing (ES) was completed by PerkinElmer in 2013, which first revealed the novel compound heterozygous variants in *POLR3D* (NM_001722.3): a missense variant (c.541C > T, p.P181S) and an intronic splice site variant (c.656-6G > A, p.?). To further investigate the cause of disease, proband ES was repeated on a research basis in 2022 using an updated sequencing platform, which iteratively confirmed the presence of biallelic variants in *POLR3D*, without detection of other candidate variants in genes encoding subunits of Pol III, including no deletions or duplications, and no detection of candidates in other leukodystrophy-associated genes. Symptom-driven analysis including genes of uncertain significance was performed, with no diagnostic or partial genotype identified for the patient. Targeted Sanger sequencing and co-segregation analysis confirmed both variants were present in the affected individual, with each parent carrying one variant (Figures 2A–C).

The *POLR3D* missense variant (c.541C > T, p.P181S) is predicted by ACMG guidelines to be pathogenic, based on its absence from population databases (PM2) and *in silico* evidence of a deleterious effect (PP3). Of interest, the missense variant is proximal to a sumoylation hotspot (Figure 2D) and resides at a conserved residue (Figure 2E).

The *POLR3D* intronic variant (c.656-6G > A, p.?) is predicted to impact splicing through disruption of the wild-type donor site and activation of a cryptic acceptor site [change at acceptor site (6 bps downstream of exon 6) = −52.9% (range − 93.4% to −12.5%); Human Splice Finder; https://hsf.genomnis.com/] (21). Moreover, the intronic variant is predicted to result in abnormal exon 7 skipping, which would lead to a premature termination codon (p.V219Gfs*13), predicted to cause loss of function due to a truncated or absent product via nonsense mediated decay. Of the 17 genes encoding Pol III subunits, *POLR3D* is the fifth gene to be associated with a leukodystrophy phenotype and the sixth gene to be associated with a genetic disease (Figure 2F) (22).

### *POLR3D* mutant fibroblasts show altered expression of Pol III subunits and Pol III targets

**Figure 3:**
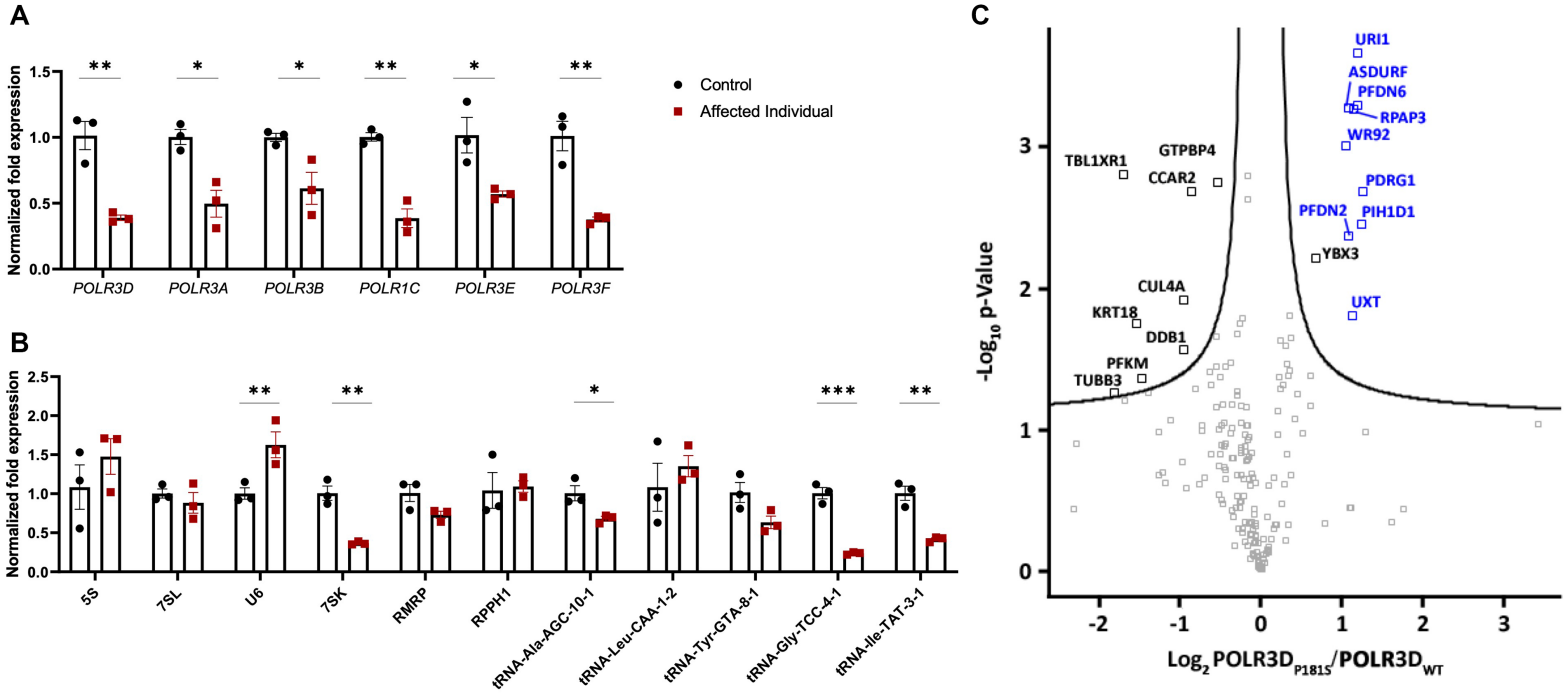
Molecular and complex assembly level implications of pathogenic variants in *POLR3D*. **(A)** RT-qPCR analysis of RNA derived from patient-derived fibroblasts and an age- and sex-matched control (*n* = 3) demonstrating significantly decreased mRNA expression of all Pol III subunits analyzed (e.g., *POLR3A*, *POLR3B*, *POLR1C*, *POLR3D*, *POLR3E*, and *POLR3F*) in the affected individual. **(B)** RT-qPCR analysis of Pol III transcripts reveals a differential impact of biallelic pathogenic variants on Pol III transcription, including significantly increased expression of *U6* RNA and significantly decreased expression of *7SK* RNA, tRNA-Ala-GTA-8-1, tRNA-Gly-TCC-4-1, and tRNA-Ile-TAT-3-1 and a trend toward a decrease in tRNA-Tyr-GTA-8-1. RT-qPCR data represent mean ± SEM normalized fold expression after normalizing to reference genes *TFRC* and *PGK1*. ^*^p < 0.05, ^**^p < 0.01, ^***^p < 0.001, two-sided Student’s *t*-test. **(C)** Volcano plot illustrating the log_2_ ratio between POLR3D p.P181S and POLR3D WT proteins (*n* = 3). PAQosome subunits are marked in blue and all other proteins that cleared the False Discovery Rate (FDR) threshold of 0.05 are marked in black.

To determine the molecular impact of these *POLR3D* variants on Pol III transcription, RT-qPCR was performed on RNA extracted from primary fibroblasts of the patient and age/sex-matched control cells. RT-qPCR assessment revealed a significant decrease in *POLR3D* RNA-level expression, as well as a significant decrease in expression of several other Pol III subunits (i.e., *POLR3A*, *POLR3B*, *POLR1C*, *POLR3E*, and *POLR3F*; Figure 3A). Assessment of Pol III transcript expression revealed a significant decrease in *7SK* RNA. In contrast, *U6* spliceosomal RNA was significantly increased relative to the control. The Pol III transcripts *5S*, *7SL*, *RPPH1*, and *RMRP* were not significantly altered. Upon analysis of distinct tRNAs, expression levels of most analyzed tRNAs were reduced. Relative to the control, tRNA-Ala-AGC-10-1, tRNA-Gly-TCC-4-1, and tRNA-Ile-TAT-3-1 showed a significant decrease in expression, with tRNA-Tyr-GTA-8-1 trending toward a decrease but not reaching statistical significance (Figure 3B).

### The POLR3D p.P181S missense variant impacts the interaction of Pol III with its chaperone complex

The missense mutation seen here resides in an unstructured region of the RPC4 protein (23). In line with this, affinity purification coupled to mass spectrometry (AP-MS) analysis revealed that all Pol III subunits were detected (Supplementary Table S2) and that the missense variant did not impact interactions between RPC4 and its partnering Pol III complex subunits. Nonetheless, nearly all subunits (9/12) of the PAQosome (i.e., the chaperone complex that regulates Pol III) were significantly increased, suggestive of prolonged interaction of the complex with the p.P181S mutant Pol III complex (Figure 3C).

## Discussion

In this study, we report the first individual with a clinical and radiological diagnosis of POLR3-related leukodystrophy caused by biallelic pathogenic variants in *POLR3D*. The individual had neurological and non-neurological features characteristic of POLR3-related leukodystrophy including motor signs (e.g., cerebellar), cognitive involvement and dysphagia, for the former, and hypodontia, hypogonadotropic hypogonadism, and dysmorphic facial features, for the latter. Further, her MRI pattern was consistent with POLR3-related leukodystrophy, as described above. Based on this clinical and neuroimaging overlap, she had initially been clinically diagnosed with 4H leukodystrophy despite the absence of variants in Pol III genes known to cause this disorder (e.g., *POLR3A*, *POLR3B*, *POLR1C*, and *POLR3K*). Overall, the individual’s disease course was consistent with the typical presentation of later-onset POLR3-related leukodystrophy, involving disease onset in school years with cognitive impairment, later followed by motor features, with or without non-neurological features (3, 5).

*POLR3D* encodes the RPC4 subunit of Pol III, which has previously been implicated in transcription initiation and termination (23). Interestingly, RPC4 is known to be affected by some POLR3-related leukodystrophy variants in the *POLR3B* gene, which cause destabilization of RPC4/RPC5 dimer assembly (24). Further, RPC4 is distinguished as a source of Pol III complex regulation due to its numerous sumoylation sites (25). Sumoylation is a type of post-translational modification conserved from yeast to human, and within RPC4 specifically, sumoylation is thought to be a mechanism of negative regulation for the Pol III complex (25). Indeed, sumoylation of Rpc53, the yeast homolog of RPC4, has been shown to induce ubiquitylation of the complex, followed by Cdc48 segregase-mediated disassembly and ultimately, proteasomal degradation (25). The proximity of the p.P181S missense variant identified here to a sumoylation hotspot in PRC4 is perhaps relevant to the pathogenicity of this variant (23).

Upon RNA-level expression analysis of the affected gene, we found *POLR3D* to be significantly decreased. To our surprise, when we analyzed additional genes encoding Pol III subunits, these were likewise significantly decreased. Pathogenic variants in genes encoding Pol III subunits have previously been shown to be hypomorphic, and studies have demonstrated reduced protein expression of the affected subunit (9, 26). We propose that the decrease in expression of these genes could result from a compensatory mechanism. As Pol III works as an enzymatic complex, the stoichiometric balance of each subunit is of interest. In the case of a decrease in one Pol III subunit, we suggest that the transcription of non-affected Pol III genes may be dampened, as these subunits would otherwise be found in excess. However, as RNA-level expression is not a faithful representation of protein-level expression, additional work is required to support this hypothesis (27).

Functionally, Pol III plays a crucial cellular role in the expression of various non-coding RNAs. As such, we sought to assess how the variants in *POLR3D* may impact Pol III transcription. Expression of Pol III-transcribed non-coding RNAs in a diseased context is variable in the literature, which may be in part due to a range of cell types being analyzed in different studies (11, 28–30). Certain Pol III transcripts, classified as Type 3, have upstream promoters reminiscent of those used by Pol II genes, with previous work demonstrating that at least some Type 3 genes have promoters occupied by Pol II, raising the possibility of compensatory transcription (8, 31, 32). As U6 fits into this Type 3 category, this could explain why an increase in *U6* is seen. Albeit, *RMRP* and *RPPH1* likewise fit into this Type 3 category, and were not shown to be significantly altered. The *5S* and *7SL* have also shown variable changes in expression when analyzed in different cell types from individuals with POLR3-related leukodystrophy that have distinct genotypes and disease severities, while we did not see significant changes in either (11, 28, 30). The challenges associated with assessing tRNAs using techniques such as RT-qPCR, in part due to their short sizes and depth of post-transcriptional modifications, have often precluded their analysis (33). Here, we were able to assess five distinct tRNAs and found three to be significantly decreased. We likewise identified a significant decrease in the Pol III transcript *7SK*, an indirect inhibitor of mRNA production.

To explore how the missense variant in *POLR3D* might impact the assembly and/or maturation of the Pol III complex, we performed an AP-MS analysis of the p.P181S mutant compared to WT, which revealed an enrichment of the PAQosome complex with the p.P181S mutant. As the PAQosome normally interacts rapidly with newly synthesized POLR3A and is not enriched in fully assembly complexes, these results suggest that the p.P181S *POLR3D* variant might perturb Pol III complex maturation, presumably contributing to the pathogenicity of this variant (16).

In summary, we describe the first case of POLR3-related leukodystrophy caused by biallelic pathogenic variants in *POLR3D*, expanding the genetic spectrum of this hypomyelinating leukodystrophy and further supporting proper Pol III function as critical for neurodevelopment and myelination. Finally, we identify how these pathogenic variants impact enzymatic complex maturation and transcriptional activity of Pol III to provide further insight into disease mechanisms.

## Data availability statement

The datasets presented in this article are not readily available because of ethical and privacy restrictions. Requests to access the datasets should be directed to the corresponding author.

## Ethics statement

The studies involving humans were approved by Montreal Children’s Hospital and the McGill University Health Center Research Ethic Boards (11-105-PED, 2019-4972) and the Children’s Mercy Institutional Review Board (study #11120514). The studies were conducted in accordance with the local legislation and institutional requirements. Written informed consent for participation in this study was provided by the participants' legal guardians/next of kin. Written informed consent was obtained from the individual(s) for the publication of any potentially identifiable images or data included in this article.

## Author contributions

JM: Conceptualization, Data curation, Formal analysis, Writing – original draft. SP: Data curation, Formal analysis, Writing – review & editing. MP: Data curation, Formal analysis, Writing – review & editing. LT: Data curation, Writing – review & editing. KG: Data curation, Writing – review & editing. CP: Data curation, Writing – review & editing. AP: Data curation, Writing – review & editing. TP: Data curation, Writing – review & editing. IT: Data curation, Formal analysis, Writing – review & editing. BC: Data curation, Formal analysis, Writing – review & editing. GB: Conceptualization, Data curation, Formal analysis, Supervision, Writing – original draft.
